# BRIDGE: a multi-stakeholder workstream focused on assessing new clinical endpoints in ophthalmology clinical trials [Building Research Innovations and Developing Global Endpoints]

**DOI:** 10.1038/s41433-025-03832-z

**Published:** 2025-07-01

**Authors:** The BRIDGE Study Group, Sobha Sivaprasad, Anat Loewenstein, Angela Carneiro, Edoardo Midena, Francesco Bandello, Jan H. Terheyden, Jean-François Korobelnik, Javier Zarranz-Ventura, Francesco Viola, Marion R. Munk, Nicole Eter, Paolo Lanzetta, Richard Gale, Stela Vujosevic, Tunde Peto, Francesco Faraldi, José Cunha-Vaz, Robert P. Finger, Antonia Joussen, Daniel Pauleikhoff, Florent G. Revel, Nadia Zakaria, Thomas Fussaro, Ine de Goeij, Sharon Bakalash, Andrew Want, Daniel Urban, H. Nida Sen, Alyson Berliner, Carl-Gustav Olsen Glittenberg, Anke Webler-Messenger, Jennifer Lynn Pluim, Michael Tolentino, David Callanan, Caroline Baumal, Reza Safaei, Rafiq Hasan, Muhammad Ali Memon, Michelle Sylvanowicz, Joao Carrasco, Natalia Rodellar Sasot, Thomas F. Miller, Laila Narouz-Ott, Silvia Specker, Fabio Baschiera

**Affiliations:** 1 NIHR Moorfields Biomedical Research Centre, Moorfields Eye Hospital, London, UK; 2 Department of Ophthalmology, Tel Aviv Medical Center, Tel Aviv, Israel; 3 Tel Aviv University, Tel Aviv, Israel; 4 Department of Surgery and Physiology, Faculty of Medicine of The University of Porto, Porto, Portugal; 5 ULS Sao Joao, Porto, Portugal; 6 Department of Ophthalmology, University of Padova, Padova, Italy; 7 IRCCS Fondazione Bietti, Rome, Italy; 8 Department of Ophthalmology, Vita-Salute University, Milan, Italy; 9 IRCCS San Raffaele Scientific Institute, Milan, Italy; 10 Department of Ophthalmology, University Hospital Bonn, Bonn, Germany; 11 CHU Bordeaux, Service d’ophtalmologie, Bordeaux, France; 12 University Bordeaux, INSERM, BPH, Bordeaux, France; 13 Hospital Clínic de Barcelona, Barcelona, Spain; 14 Universitat de Barcelona, Barcelona, Spain; 15 Fondazione IRCCS Ca’ Granda Ospedale Maggiore Policlinico, Milan, Italy; 16 Department of Clinical Science and Community Health, University of Milan, Milan, Italy; 17 Gutblick AG, Bern, Switzerland; 18 University Hospital of Bern, Bern, Switzerland; 19 Department of Ophthalmology, University of Münster, Münster, Germany; 20 Department of Medicine - Ophthalmology, University of Udine, Udine, Italy; 21 York Teaching Hospital NHS, York, UK; 22 Department of Biomedical, Surgical and Dental Sciences, University of Milano, Milan, Italy; 23 Eye Clinic IRCCS MultiMedica, Milan, Italy; 24 Centre for Public Health, Queen’s University Belfast, Belfast, UK; 25 Azienda Ospedaliera Ordine Mauriziano di Torino, Turin, Italy; 26 Faculty of Medicine, University of Coimbra, Coimbra, Portugal; 27 Association for Innovation and Biomedical Research on Light and Image, Coimbra, Portugal; 28 Department of Ophthalmology, Medical Faculty Mannheim, University of Heidelberg, Mannheim, Germany; 29 Charité – University Medicine Berlin, Berlin, Germany; 30 Department of Ophthalmology, St. Franziskus Hospital, Münster, Germany; 31 Novartis, Translational Medicine, Cambridge, MA USA; 32 Astellas, Addlestone, UK; 33 Sanofi, Cambridge, MA USA; 34 Regeneron, Tarrytown, NY USA; 35 Boehringer Ingelheim, Ingelheim, Germany; 36 Aviceda, Cambridge, MA USA; 37 Apellis, Waltham, MA USA; 38 Complement TX, London, UK; 39 Bayer, Basel, Switzerland

**Keywords:** Medical research, Neuroscience

## Introduction

Differences in Health Authority (HA) decisions between the Food and Drug Administration (FDA) and European Medicines Agency (EMA) are leading to unequal access to new medical treatments, with European patients experiencing delays in access to innovative ophthalmic therapies relative to other first-world Nations.

**Fig. 1:**
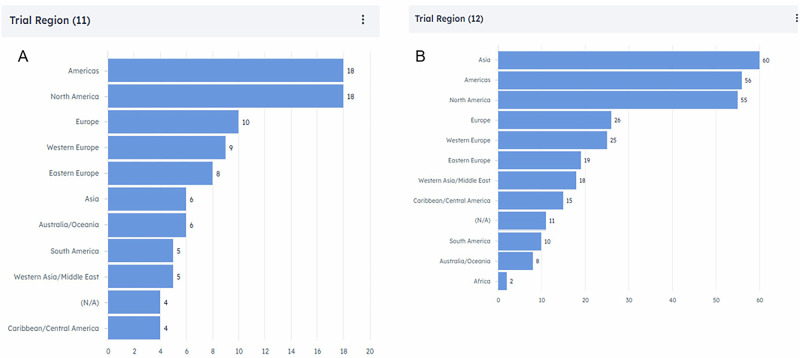
Clinical Trials in AMD and DR/DME by Geographical Region. Source: Trialtrove® | Citeline 2024: Recently completed, on-going or planned registrational trials in age-related macular degeneration (AMD) (**A**) and diabetic retinopathy (DR)/diabetic macular oedema (DMO) (**B**) by geographical region (Accessed data AMD 2024-11-15 and DR/DME 2024-11-26; adapted to BRIDGE focus).

A key challenge is the lack of clear EU guidelines on clinical endpoints for retinal disease indications, which creates uncertainty for Sponsors and may contribute to the preference for conducting ophthalmology trials outside the EU. The competitive alternatives offered by the United States and Asia (Fig. 1) [1] further widen the gap in treatment options between regions, highlighting the need to address these issues to improve access to advanced retinal treatments in Europe.

According to the U.S. Census Bureau, 8.5% of the global population was aged 65 years or older in 2016. By 2050, this proportion is expected to double to 16.7%, representing approximately 1.6 billion people worldwide. Europe and the Asia-Pacific region are expected to be the center of aging population [2]. This significant growth in the older age group population is likely to be accompanied by a corresponding rise in the prevalence of retinal disorders. Additionally, the global diabetes prevalence in 2019 is estimated to be 9.3% (463 million people), rising to 10.2% (578 million) by 2030 and 10.9% (700 million) by 2045, highlighting the growing burden of diabetes-related complications, including diabetic retinopathy (DR) [3]. Studies on the impact on healthcare costs of late-stage age-related macular degeneration (AMD), especially in relation to the developing demography, are lacking [4]. It is estimated that the total cost by 2030 associated to glaucoma, diabetic retinopathy (DR), diabetic macular oedema (DMO), AMD and high myopia (HM) could amount to 99.8 billion euros. Direct non-healthcare costs account for the largest item (44%), followed by loss of productivity costs (38%), and direct healthcare costs (18%) [5].

This report summarizes the outcomes of two foundational expert workshops aimed at addressing these clinical research issues, intended to foster collaboration and establish a harmonized consensus around ophthalmological clinical endpoints.

## The BRIDGE workstream

Established in late 2024, the BRIDGE workstream was initially launched by academia and industry experts (with the goal of broadening participation to patient advocates, HA executives and Health Technology Assessments) as a call to action to initiate collaboration and advance the development of standardized clinical endpoints in ophthalmology, with focus on retinal disease indications. For this purpose, two expert workshops were held aside the conferences, EURETINA (Barcelona, Spain, September 2024) and FLORetina (Florence, Italy, December 2024). Workshop attendees proposed focusing on the following three so-called macro-areas of disease, based on underlying pathological mechanisms rather than specific indications:

1. Ischemic diseases: with focus on DR and occlusive retinal conditions.
2. Atrophic diseases: with focus on AMD.
3. Other conditions: e.g., neurodegenerative

This categorization enables cross-referencing of endpoints of underlying cause and a more streamlined approach to regulatory evaluations.

It was noted that FDA accepts anatomical endpoints for ophthalmological products when a clear relationship between structural and functional endpoints is established, as outlined in its specific guidance [6, 7]. In the EU, there seems to be a preference for functional endpoints over anatomical ones in ophthalmology, emphasizing the need for clinically meaningful evidence. To gain acceptance in the EU, anatomical endpoints must be rigorously validated with prospective data to demonstrate their clinical relevance, leaving the presentation of the evidence with the compound’s dossier. An observation from recent regulatory decisions evidences the need to achieve consensus for earlier efficacy endpoints for (slowly) progressing and, in the end, debilitating serious diseases.

A roadmap for the BRIDGE initiative was outlined, beginning with a comprehensive literature review to analyze existing data, identify gaps, and guide future publications. This approach aims to: (1) avoid ad-hoc analysis bias to achieve consensus, (2) facilitate interactions with regulators on a common and solid basis, and (3) remain outside the competitive space by excluding references to any commercialized or in-development products. Achieving this goal requires a careful characterization of the pathways associated with disease progression in each macro-area, utilizing published data to support these objectives.

Following this, additional efforts may include: conduct of global patient registry search, data repository development; establishing a prospective trial or collecting new data to build a robust repository; and data analysis using artificial intelligence (AI) algorithms, leveraging AI to analyze large imaging datasets from available research-standard resources like the Innovative Medicines Initiative (IMI) MACUSTAR [8] and the Mary Tyler Moore Vision Initiative [9]. These latter steps could further strengthen the initiative as it progresses.

BRIDGE envisions engaging relevant patient advocacy associations to ensure the relevance of the work to those who will benefit was emphasized, alongside the incorporation of patient-reported outcomes and quality-of-life assessments capturing the real-world impact of treatments. Developing a standardized questionnaire reflecting patient priorities, such as the effects of visual impairments on daily living activities, was proposed as a crucial element for HA submissions. Since this initiative is focusing on ‘pre-competitive’ topics, the workshop attendees emphasized the importance of active participation by regulatory scientists from global HAs.

The initial step involves grouping the Workstream’s participants according to their areas of expertise within the macro-areas and identifying structural determinants of atrophic and/or ischemic disease progression, as well as functional vision loss, based on published natural history data. Further expertise in structural analysis may be contributed by device manufacturing companies.

## Conclusion

The discussions from these workshops reflected a strong consensus on the need to establish standardized clinical endpoints for ophthalmological research and product development, ideally harmonized across global HAs. By categorizing diseases into macro-areas and adopting a multidimensional approach to endpoints, this initiative seeks to align the EU’s regulatory framework with emerging international standards. Collaboration among academia, industry, HAs, and patient organisations will be essential to achieving this goal. Next steps include the creation of two parallel workstreams, for both atrophic and ischemic macro-areas, evaluating published evidence to assess: 1. Disease staging; 2. Diagnostic methods; 3. Disease progression indicators; 4. Structure-function correlations; 5. Indicators of patient-relevance.
